# Experimental demonstration of a reconfigurable electro-optic directed logic circuit using cascaded carrier-injection micro-ring resonators

**DOI:** 10.1038/s41598-017-06736-5

**Published:** 2017-07-25

**Authors:** Yonghui Tian, Zilong Liu, Huifu Xiao, Guolin Zhao, Guipeng Liu, Jianhong Yang, Jianfeng Ding, Lei Zhang, Lin Yang

**Affiliations:** 1 Institute of Microelectronics and Key Laboratory for Magnetism and Materls of MOE, School of Physical Science and Technology, Lanzhou University, Lanzhou, 730000 Gansu China; 2 State Key Laboratory on Integrated Optoelectronics, Institute of Semiconductors, Chinese Academy of Sciences, P.O. Box 912, Beijing, 100083 China

## Abstract

We experimentally demonstrate a reconfigurable electro-optic directed logic circuit which can perform any combinatorial logic operation using cascaded carrier-injection micro-ring resonators (MRRs), and the logic circuit is fabricated on the silicon-on-insulator (SOI) substrate with the standard commercial Complementary Metal-Oxide-Semiconductor (CMOS) fabrication process. PIN diodes embedded around MRRs are employed to achieve the carrier injection modulation. The operands are represented by electrical signals, which are applied to the corresponding MRRs to control their switching states. The operation result is directed to the output port in the form of light. For proof of principle, several logic operations of three-operand with the operation speed of 100 *Mbps* are demonstrated successfully.

## Introduction

Silicon photonics has attracted more and more attention in scientific community due to its natural advantages such as complementary metal-oxide-semiconductor-compatible (CMOS) fabrication process, high transmission speed, low latency, and parallel processing, etc^1–6^. Currently, silicon photonics has achieved great development in many fields, and various silicon-based optical devices have been demonstrated successfully such as filters^7–9^, routers^10–12^, lasers^13–17^, multiplexers^18–22^, sensors^23–27^, electro-optic modulators^28–38^ and optical logic devices^39–45^, etc. Being a kind of basic devices in the area of silicon photonics, micro-ring resonator (MRR) is widely used in high-performance computing and optical information processing due to its unique advantages such as sharp resonances for wavelength selectivity, compact size, low consumption, and large-scale integration.

Electro-optic directed logic is a novel paradigm which employs the optical switch networks to carry out Boolean logic operations, and the electrical signals regarded as logic operands are applied to the optical switches to control their switching states^46–48^. The operands of the Boolean logic operations determines the state of each optical switch whose operation is independent to the others in the optical switch network, and all optical switches can perform their operations almost simultaneously. Since the delay time of each switch will not accumulate, and the final operation result is directed to the output port in the form of light, the overall latency of the logic circuit is very low. In addition, as it is known to all, the advantage of electrical signal lies in its convenience of control, and that of the light signal is its adaptability to operation derived from the high propagation speed. The electro-optic directed logic combines the advantages of both electrical and light signals since its control signal is electron and operation signal is photon. Therefore, electro-optic directed logic is a highly appealing candidate for future high speed, bit-rate optical computing, networking system and highly integrated on-chip photonic system. Note that for the directed logic scheme, the logic operands are electrical signals, and the operation results are output in the form of light. The output signals (light signals) must be converted into electrical signals to drive the next level. Therefore, the integrated photo-detector is needed for the scalability. However, the most desired application occasion of directed logic is in on-chip optical network, in where the operation results can be applied in next level directly rather than needed to be converted into electrical signals, and in this case, directed logic can provide ultrafast network routing functions that enable highly efficient packet-switched interconnections for high-performance computing. In a word, for some special application occasion where the operation results in the form of light can be directly applied in the next level, directed logic has more advantages over electrical logic by taking advantage of fast and low-loss propagation of light in a highly integrated on-chip photonic system.

Reconfigurable electro-optic directed logic plays a key role in optical information processing. Compared to most of electro-optic directed logic circuits, reconfigurable electro-optic directed logic circuit can perform any combinatorial logic operation, which can be applied in many occasions such as optical computing, packet routing, etc. For proof of concept, we have successfully demonstrated a reconfigurable electro-optic directed logic circuit which can perform any combinatorial logic operation with four-operand at the operation speed of 10 *kbps* based on the silicon thermo-optic effect in our previous work^42^. However, with the growing demand of high speed optical computing, the drawback of the device in ref. 42 has appeared. In this paper, we report a higher speed reconfigurable logic circuit using cascaded carrier-injection MRRs. Although relatively faster response time of reconfigurable electro-optic directed logic circuits has been reported^43, 44^, the Multiplexer (MUX)/Demultiplexer (DEMUX) and computing functions are achieved simultaneously by the proposed logic circuit, where the MUX/DEMUX are integrated with computing elements. Table 1 summarizes the comparison of various reconfigurable electro-optic directed logic circuits. In comparison, our logic circuit does not need additional MRRs to achieve MUX/DEMUX functions. Therefore, it is more compact than refs 43 and 44. We design and fabricate the logic circuit on Silicon-Oxide-Insulator (SOI) substrate using the standard complementary metal-oxide-semiconductor (CMOS) process, and several logic operations with the operation speed of 100 *Mbps* are finally demonstrated successfully.

**Table 1:** A comparison of various reconfigurable electro-optic directed logic circuits.

Structure	Extra MUX/DEMUX	Number of Operands	MRRs required in reconfigurable two-operand operation	Modulation scheme	Operation speed
2 × 2 Switches array^*a*^	YES	4	7	Forward biased PIN junction	~500 *Mb*/*s*
2 × 2 Switches array^*b*^	YES	4	7	Reverse biased PN junction	3 *Gb*/*s*
1 × 4 Switches array^*c*^	NO	4	2	Micro-heater	10 *Kb*/*s*
1 × 3 Switches array^*d*^	NO	3	2	Forward biased PIN junction	100 *Mb*/*s*

^*a*^Taken from ref. 43.

^*b*^Taken from ref. 44.

^*c*^Taken from ref. 42.

^*d*^Taken from this work.

## Results

### Device working principle, design and fabrication

**Figure 1:**
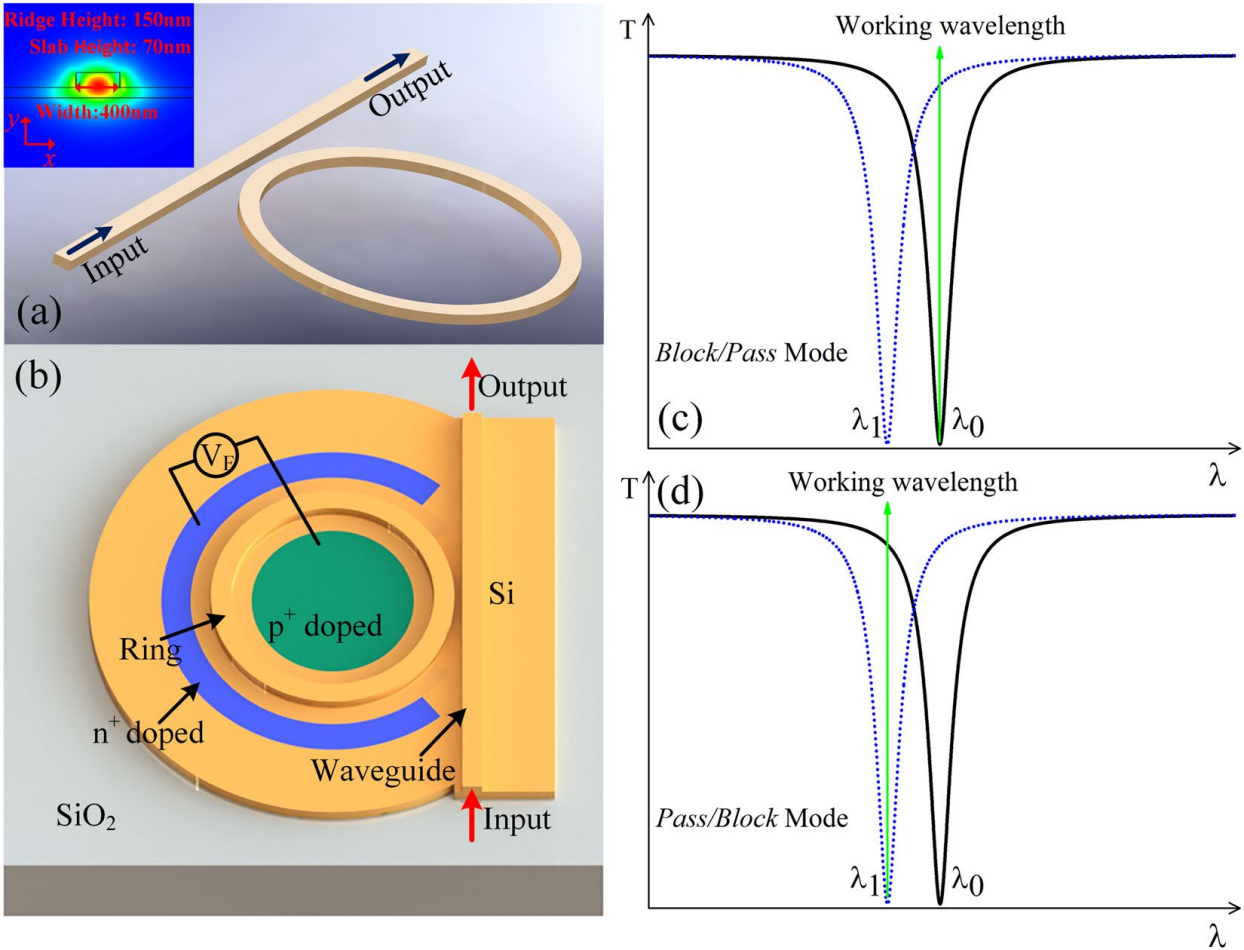
(**a**) Single MRR with one waveguide (inset: the cross-section of the device including the quasi-TE fundamental mode and dimensions of waveguide), (**b**) a tunable MRR-based optical switch with a PIN junction embedded in the ring, (**c**) the transmission spectra of the optical switch in *block*/*pass* mode (the status of the optical switch is from OFF to ON) for light with the working wavelength, black line is the spectra when the logic signal is ‘0’, blue line is the spectra when the logic signal is ‘1’, *λ*_0_ marks the resonant wavelength without modulation, *λ*_1_ marks the resonant wavelength after modulation, (**d**) the transmission spectra of the switch in *pass*/*block* mode (the status of the optical switch is from ON to OFF).

It is well known that any logic function can be expressed in the form of sum-of-product. For example, an arbitrary logic function *Y* can be expressed as $Y={X}_{1}+{X}_{2}+{X}_{3}\ldots \ldots {X}_{n}$, and *X*_*n*_ denotes the product of a number of variables, such as ${X}_{n}={a}_{1}{\bar{a}}_{2}{a}_{3}\ldots \ldots {a}_{n}$(${\bar{a}}_{n}$ represents the opposite logic value of *a*_*n*_). According to the logic expression, we propose a reconfigurable electro-optic directed logic circuit which can perform any combinatorial logic operation based on cascaded carrier-injection MRRs. As we know, MRR can be employed to construct the optical switch (Fig. 1(a)). In order to control the working state of the optical switch, we fabricate a PIN junction embedded around the ring (Fig. 1(b)), and thus the electrical signal can be applied to MRR through the PIN diodes to achieve a high speed modulation.

Generally speaking, there are two different operation modes for the optical switch. One is the *block*/*pass* mode, and the other is the *pass*/*block* mode. Figure 1(c,d) show the mechanisms to realize the two different operation modes of optical switch, respectively. The resonant wavelength without modulation and the resonant wavelength after modulation are represented by *λ*_0_ and *λ*_1_, respectively. When the working wavelength aligns with *λ*_0_ (see Fig. 1(c)), the switch works in the *block*/*pass* mode; while the working wavelength aligns with *λ*_1_ (see Fig. 1(d)), the switch works in the *pass*/*block* mode.

**Figure 2:**
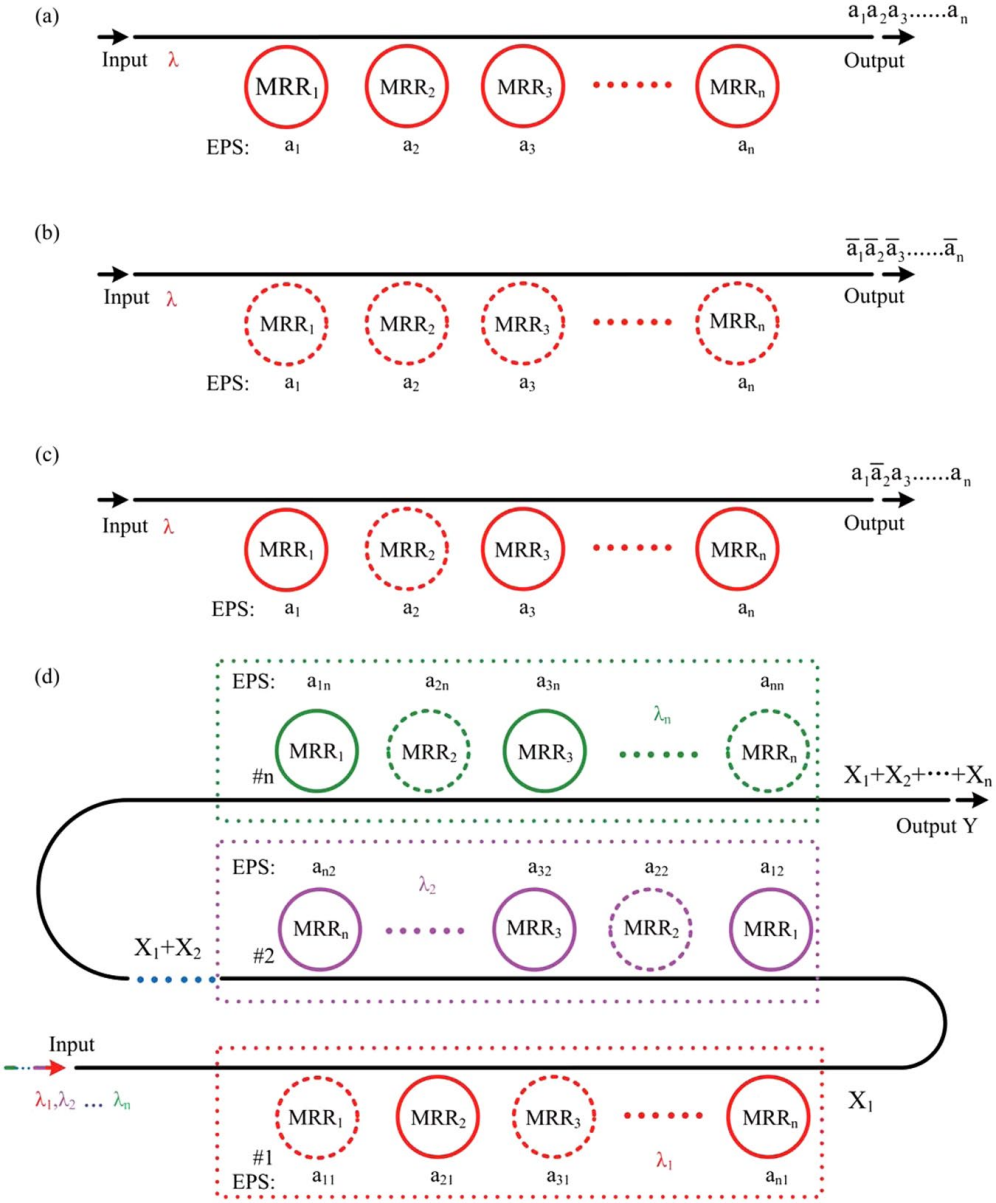
The logic circuit with (**a**) one working wavelength and all MRRs work in the *block*/*pass* mode, (**b**) one working wavelength and all MRRs work in the *pass*/*block* mode, (**c**) one working wavelength and MRRs work in the *pass*/*block* or *block*/*pass* mode, and (**d**) the proposed reconfigurable logic circuit (the solid line ring denotes MRR working in the *block*/*pass* mode, the dot line ring denotes MRR working in the *pass*/*block* mode, MRRs with the same color work at the same working wavelength, EPS: electrical pulse sequences).

The structure of the proposed logic circuit is composed of N MRRs and one waveguide, which is illustrated in Fig. 2(d). In fact, the essence of the proposed logic circuit is an optical switch array consisting of N MRR-based optical switches. Monochromatic continuous light with the working wavelength of *λ* is coupled into the input ports of logic circuits (Fig. 2(a–c)), and then the light is modulated by the electrical pulse sequences (EPS) applied to MRR_1_, MRR_2_, MRR_3_, …, MRR_*n*_, respectively. The low and high levels of EPS represent logic ‘0’ and ‘1’ in electrical domain, respectively; the low and high levels of the optical power at the output port represent logic ‘0’ and ‘1’ in optical domain, respectively. As mentioned in our previous work^42^, the logic circuit of Fig. 2(a) can perform the AND operation of *N* operands when all MRRs work in the *block*/*pass* mode. The operation result *X* can be expressed as $X={a}_{1}{a}_{2}{a}_{3}\ldots \ldots {a}_{n}$. Similarly, the logic circuit can also perform the AND operation of *N* inverse operands when all MRRs work in the *pass*/*block* mode. The operation result can be expressed as $X={\bar{a}}_{1}{\bar{a}}_{2}{\bar{a}}_{3}\ldots {\bar{a}}_{n}$ (Fig. 2(b)). The logic circuit can also perform the AND operation of *N* operands when some MRRs work in the *pass*/*block* mode and others work in the *block*/*pass* mode. For instance, we define MRR_2_ working in *pass*/*block* mode and the other MRRs working in *block*/*pass* mode in Fig. 2(c). The operation result *X* can be expressed as $X={a}_{1}{\bar{a}}_{2}{a}_{3}\ldots \ldots {a}_{n}$. In brief, if the continuous wave coupled into the logic circuit is monochromatic, the product of any *N* variables can be obtained at the output port in the form of light according to the definitions of MRR operation modes. As the circuit diagrams show in Fig. 2(d), MRRs in the logic circuit can be divided into several operation groups, where MRRs in one group work at the same wavelength. Multi-wavelength signal lights with the wavelength of *λ*_1_, *λ*_2_, ${\lambda }_{3}\ldots {\lambda }_{n}$ are coupled into the input port simultaneously, and are separated and directed into different operation groups with the Wavelength Division Multiplexing (WDM) technology. Based on the above discussions, every operation group can perform the product of any variables. The operation results of all operation groups are finally multiplexed together. Therefore, the final operation result *Y* can be expressed as $Y={X}_{1}+{X}_{2}+{X}_{3}\ldots \ldots {X}_{n}$ (*X*_*n*_ denotes the product of any variables). Note that the amount of operation groups is equal to the amount of the working wavelengths, and the amount of operands in each term is equal to the amount of MRRs in the corresponding operation group. The proposed logic circuit is similar with the Field Programmable Gate Array (FPGA), and the resonant wavelengths of unused MRRs can be shifted far from the working wavelengths of the circuit through thermal tuning.

**Figure 3:**
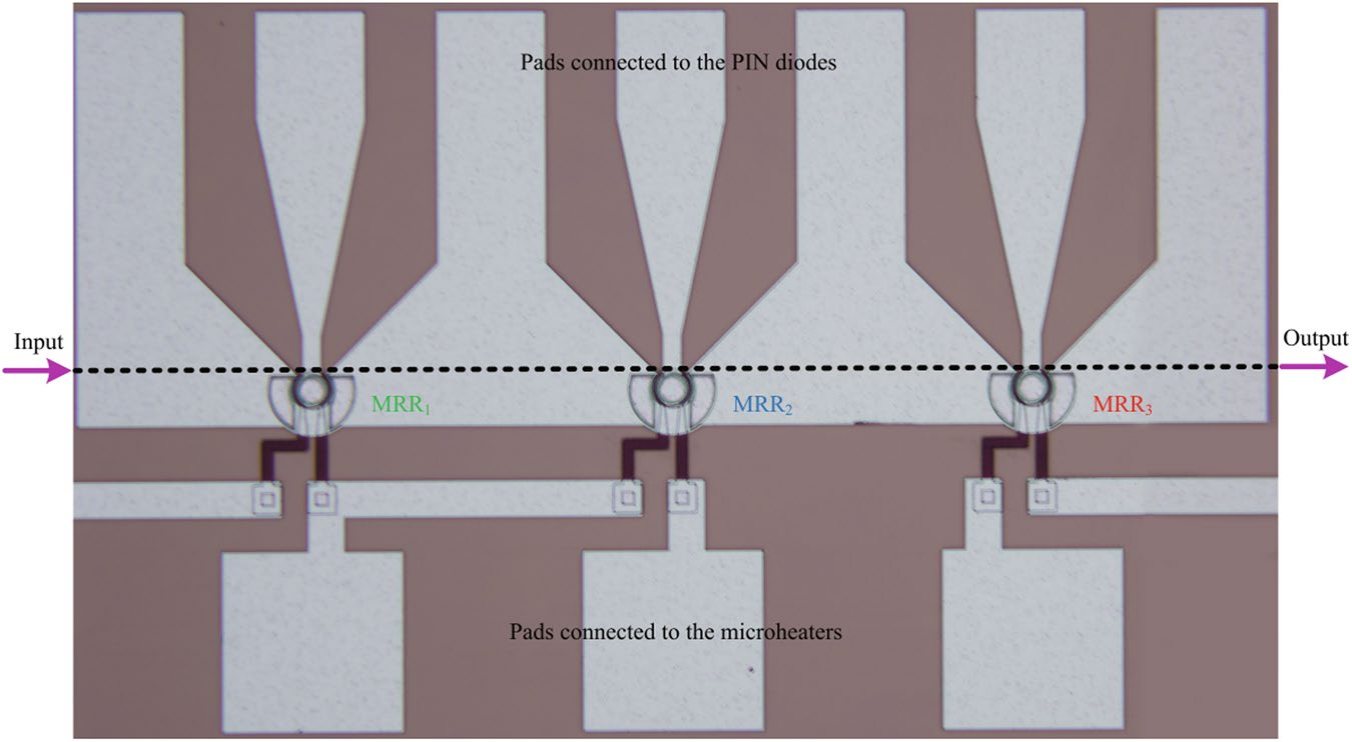
Microscope image of the fabricated reconfigurable electro-optic directed logic circuit based on silicon MRRs.

For a proof of principle, a reconfigurable electro-optic directed logic circuit consisting of three electro-optic tunable MRRs is fabricated on 8 in. (20.3 cm) silicon-on-insulator (SOI) wafer with 2-*μm*-buried *SiO*_2_ layer and 220-nm-top silicon layer. The microscope image of the fabricated device is shown in Fig. 3, and the efficient footprint of the fabricated device is about 400 × 1100 *μm* ^2^. The carrier-injection-based PIN modulation structure is used to achieve relatively high speed and high-efficiency modulation of the device. As we known, when the PIN junction is forward-biased, the carriers are injected into the core of the waveguide through the diffusion motion of the carriers, and then the refractive index of the waveguide is modulated. Generally, the carrier’s diffusion speed is far lower than its drift speed; therefore, the operation speed of the device based on the carrier-injection modulation scheme is lower than that’s the carrier-depletion modulation scheme. However, the number of the carrier injected into the core of the waveguide can be very large when a small forward voltage is applied to the PIN junction; therefore, the forward-biased PIN modulation scheme has higher modulation efficiency compared to the reverse-biased PN modulation scheme. For some specific application occasion in which the modulation efficiency is more desired than the operation speed, the forward-biased PIN modulation scheme is a better choice. In fact, these two modulation schemes (forward-biased PIN junction and reverse-biased PN junction) are both based on the free carrier dispersion effect, and in here, we choose the PIN modulation scheme with the simple fabrication process to verify that the free carrier dispersion effect can also be employed in our proposed device. The device is fabricated in Institute of Microelectronics (IME), Singapore using the standard commercial Complementary Metal-Oxide-Semiconductor (CMOS) process, and the fabrication process is similar to our previous works^40, 41^. See Methods for full fabrication process.

### Static Response Test

**Figure 4:**
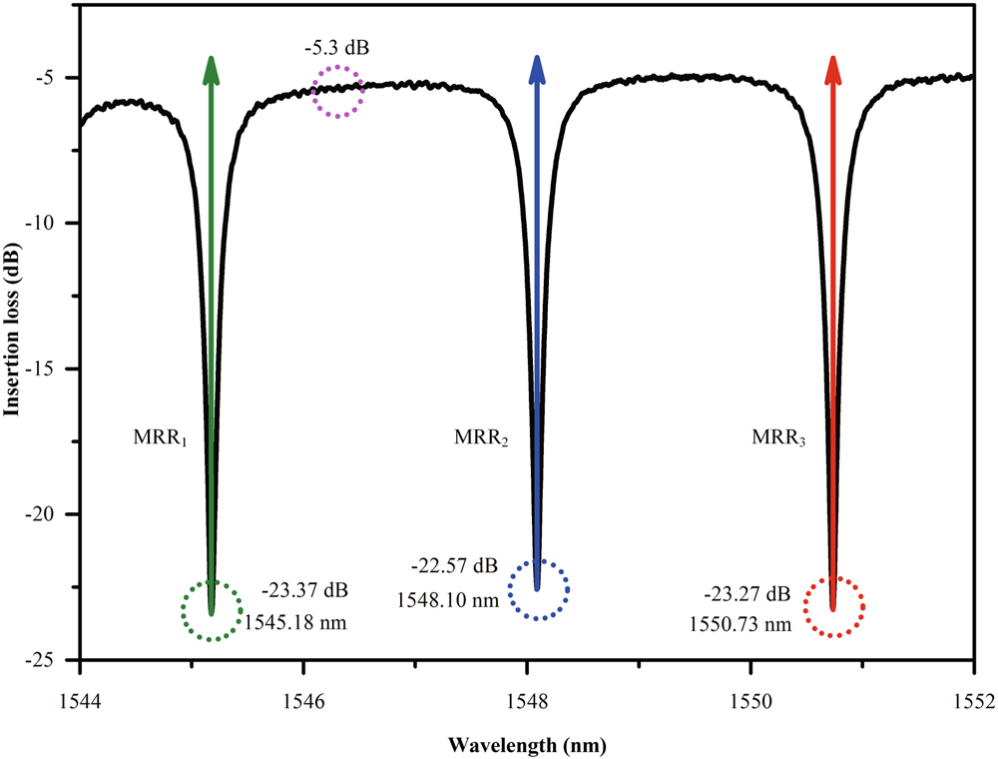
Static response spectral of the device with no voltage applied to MRRs.

**Figure 5:**
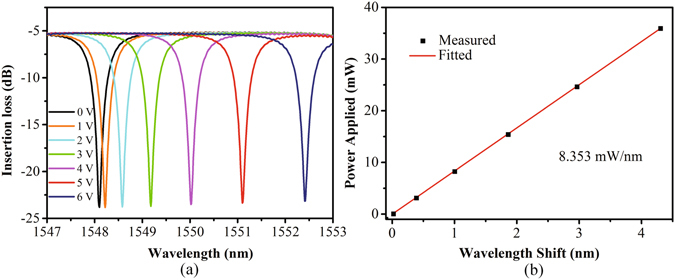
(**a**) Resonance shift of the MRR with different driving voltages, as well as (**b**) the tuning efficiency of the heater.

In order to determine the working wavelengths of the device, an amplified spontaneous emission (ASE) source and an optical spectrum analyzer (OSA) are employed to measure the static response spectra of the device. The measured static response spectra with no voltage applied to the device is shown in Fig. 4. The insertion loss is about 5.3 dB which includes 5 dB coupling loss (about 2.5 dB for each end face) and 0.3 dB transmission loss; the resonant wavelengths of three cascaded MRRs are 1545.18 nm, 1548.10 nm, 1550.73 nm, respectively. The extinction ratio of each MRR is nearly equal (18 dB), and the quality factor (Q factor) of each MRR is approximately 5,000. For carrier-injection modulation, high Q factors are desirable for low-voltage and low-power operation. High Q factor means small 3-dB bandwidth to some extent; therefore, small carrier concentration change can result in enough extinction ratios. However, the trade-off of Q factor should be taken into account since the higher the Q factor is, the more sensitive the MRR will be to the change of environmental temperature. In addition, a high Q factor means long photon lifetime, which will further limits the working speed of the MRR. Therefore, a moderate Q factor of 5,000 is suitable for the device demonstrated in this paper. Consequently, we use three electro-optic tunable MRR with suitable parameters to demonstrate the reconfigurable logic operations. Note that the Ω-shape heaters are integrated on the top of MRRs in order to control their resonant wavelength accurately, and the thermal tuning response spectral is shown in Fig. 5(a), from which we can see that the MRR’s resonant wavelength occurs red-shift with the increasing of applied voltage. The thermal tuning efficiency is about 8.353 mW/nm (as shown in Fig. 5(b)), which can be greatly improved by introducing air trench around MRR^49^. In addition, air trench can also decrease the crosstalk between adjacent MRRs and improve the thermal stabilization of MRR. Therefore, air trench issue is a main consideration for the optimization design of the device in future.

### Dynamic Response Test

**Figure 6:**
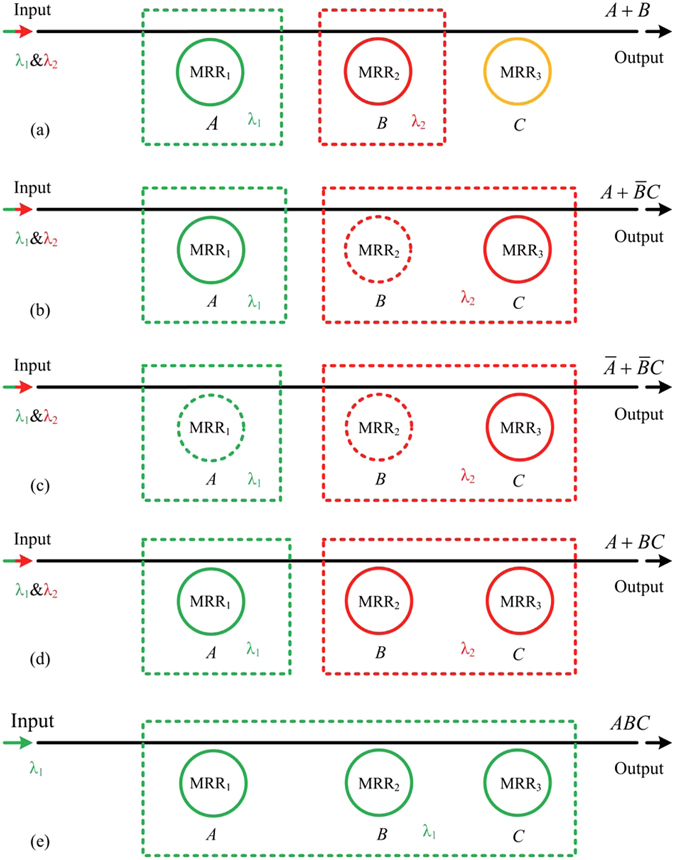
Logic circuit diagrams of the device for different three-input logic operations (the solid line ring denotes MRR works in the *block*/*pass* mode, the dot line ring denotes MRR works in the *pass*/*block* mode, MRRs with the same color work at the same working wavelength).

As we all know, there are many different logic operations for the three-operand. However, for the purpose of proving the principle, only a few typical logic operations of them are demonstrated here. Firstly, three MMRs are divided into two operation groups, and the first group has one MRR while the second group has two MRRs (Fig. 6). The first group is composed of MRR_1_, and the working wavelength is chosen as *λ*_1_. The second group is composed of MRR_2_ and MRR_3_, and the working wavelength is chosen as *λ*_2_. The different operation modes for MRR are defined to perform different logic operations. For example, two lasers with the wavelengths of *λ*_1_ and *λ*_2_ are coupled into the input port of the device simultaneously, if the MRR_1_ is controlled by operand *A* in the *pass*/*block* mode, the MRR_2_ is controlled by operand *B* in the *pass*/*block* mode and the MRR_3_ is controlled by operand *C* in the *block*/*pass* mode, and thus the device can perform the logic operation of $\bar{A}+\bar{B}C$ (Fig. 6(c)). Analogously, other logic operations can also be achieved through the definitions of different operation modes of MRR, and several typical examples are shown in Fig. 6.

**Figure 7:**
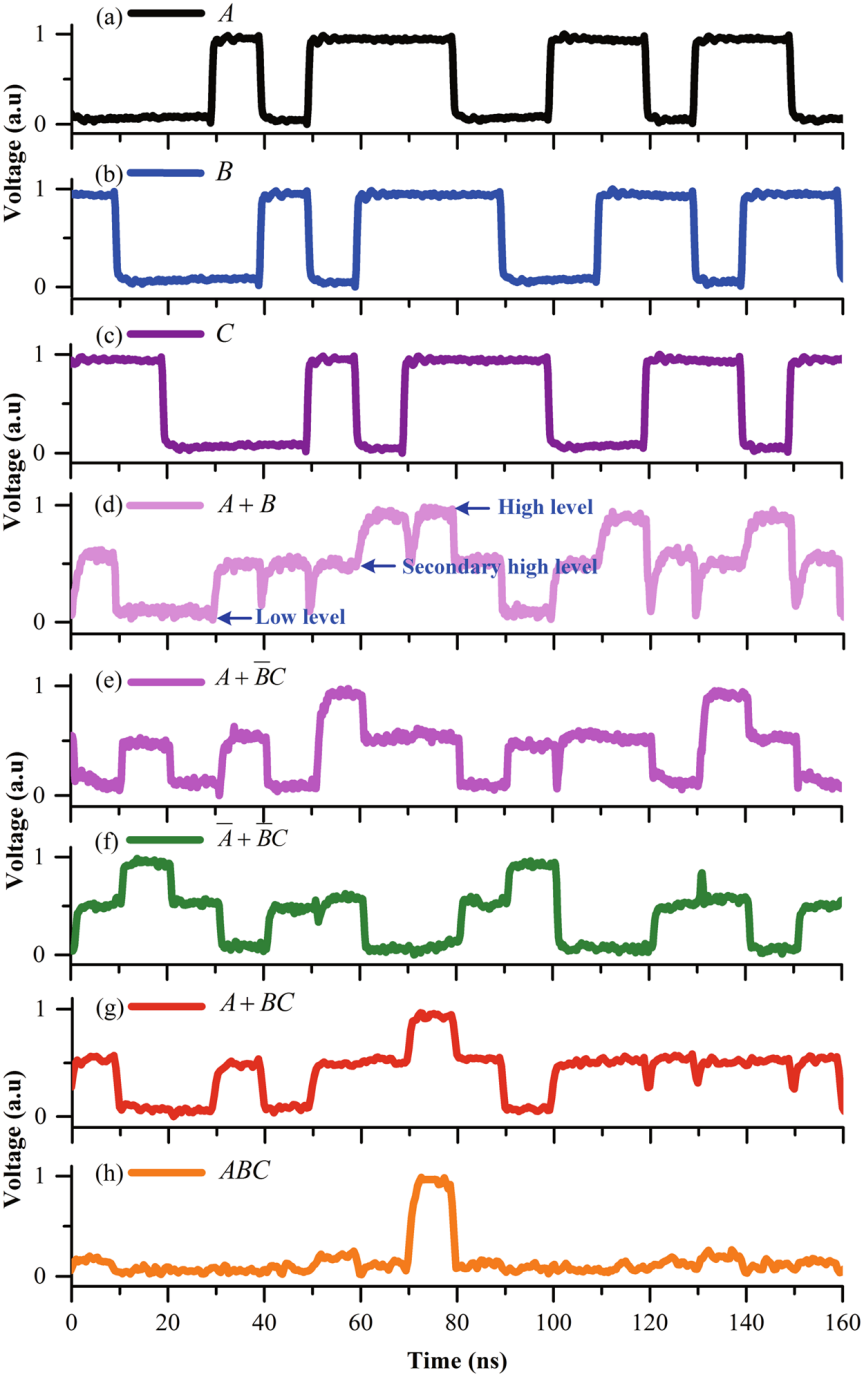
Logic operation results of the device with three-operand ((**a**–**c**) represent the operands applied to MRRs, and (**d**–**h**) represent the logic operation results).

The measured dynamic response results of the device are shown in Fig. 7. A two-channel tunable laser (TL), three arbitrary function generators (AFGs), an oscilloscope (OSC), an erbium doped fiber amplifier (EDFA), an optical filter, and a photo-detector (PD) are employed to characterize the dynamic response of the device. (Note that those facilities were only used to characterize the device in our experiment, while in practical application, they are no needed and the logical function of the device can be used just like traditional electric transistors.) Three MRRs are firstly divided into two groups. The first group is only composed of MRR_1_, and its working wavelength is *λ*_1_. The second group includes MRR_2_ and MRR_3_, and its working wavelength is *λ*_2_. Continuous monochromatic lights with the wavelengths of *λ*_1_ and *λ*_2_ from a two-channel tunable laser are simultaneously coupled into polarization controllers and then the monochromatic light with TE polarization is coupled into the device through a 2 × 1 combiner. Three pseudo-random binary sequence (PRBS) non-return-to-zero signals with the speed of 100 *Mbps* generated by the AFGs are applied to MRRs through the PIN diodes embedded around MRRs, respectively. The voltage swing of the PRBS is 1.50 *V*. The DC bias voltages are −0.06 *V* in *block*/*pass* mode and −0.20 *V* in *pass*/*block* mode for MRR_1_, and the DC bias voltages for MRR_2_ and MRR_3_ are the same, which are −0.19 *V* in *block*/*pass* mode and −0.26 *V* in *pass*/*block* mode, respectively. Therefore, the operating voltages are different in different operation mode for the same MRR, and the operating voltages are 0.69 V in logic ‘1’ state and −0.81 V in logic ‘0’ state for MRR_1_ in *block*/*pass* mode, which means the operating voltage change from 0.69 *V* to −0.81 *V* when the logic signal change from logic ‘1’ to ‘0’, vice-versa; The operating voltages are 0.55 *V* in logic ‘0’ state and −0.95 *V* in logic ‘1’ state for MRR_1_ in *pass*/*block* mode, which means the operating voltage change from 0.55 *V* to −0.95 *V* when the logic signal change from logic ‘0’ to ‘1’, vice-versa. The voltage swings and DC bias voltages are the same for MRR_2_ and MRR_3_, therefore, the operating voltages change from 0.56 *V* to −0.94 *V* for MRR_2_ and MRR_3_ in *block*/*pass* mode when the logic signals change from logic ‘1’ to ‘0’, vice-versa, and the operating voltages change from 0.49 *V* to −1.01 *V* for MRR_2_ and MRR_3_ in *pass*/*block* mode when the logic signals change from logic ‘0’ to ‘1’, vice-versa.

Finally, the output optical signal is coupled into a photo-detector, and the electrical signal transformed by the PD is fed into the OSC for observation. As proof of principle, several logic operations of three-operand with the operation speed of 100 *Mbps* are demonstrated successfully (Fig. 7). In fact, any logic operation of three-operand can be achieved by the proposed device through the alteration of the working wavelength numbers and the operation modes of optical switch. Definitely, we can increase the numbers of MRR in the logic circuit in order to achieve more complex logic operations. In a word, the proposed logic circuit can perform reconfigurable logic operations by using WDM technology. Note that although the high levels of the results are different attributed to the WDM technology, it does not affect the performance of the device since we can define the secondary high level as logic1 (Fig. 7(d)). In addition, some undesired small peaks and dips can be found in Fig. 7 resulting from the alteration of working status for the device, which has been discussed in detail in ref. 40. Note that the device can perform higher operation speed when the pre-emphasis electrical signals (logic operands) are used to reduce MRRs’ response time^30^. In fact, the modulation of the proposed device is to change the MRR’s resonant wavelength, in other word, all modulation schemes which can change the refractive index of the waveguide can be employed in the design of the device. Therefore, other advanced modulating schemes such as the carrier-depletion modulation and the electric field effects can also be employed to modulate the MRRs to achieve a higher speed operation of the device. However, the modulation efficiency for the carrier-depletion modulation and the electric field effects is relatively low. In order to obtain the faster operation speed as well as higher modulation efficiency, the optimization of the device will be a potential challenge in future. The power consumption of the device is related to the specific logic operation, which means the power consumptions are different if the device performs different logic operations. The specific power consumptions of several logic operations demonstrated in the paper are given in Table 2. In fact, the supplied DC power consumption can be greatly decreased by fabricating air trench around MRR. Therefore, the total power consumption can be further decreased, which has been left for our future works.

**Table 2:** Supplied power consumption for different Boolean operations.

Boolean operations	*A* + *B*	${\boldsymbol{A}}+\bar{{\boldsymbol{B}}}C$	$\bar{{\boldsymbol{A}}}+\bar{{\boldsymbol{B}}}C$	*A* + *BC*	*ABC*
Supplied dynamic power consumption	23.26 *mW*	35.90 *mW*	36.61 *mW*	35.20 *mW*	35.20 *mW*
Supplied DC power consumption	0 *mW*	38.44 *mW*	38.44 *mW*	31.36 *mW*	97.54 *mW*
Supplied total power consumption	23.26 *mW*	74.34 *mW*	75.05 *mW*	66.56 *mW*	132.74 *mW*

## Conclusion

We report a reconfigurable electro-optic directed circuit which can implement any logic operation using cascaded MRRs, and in order to realize a high operation speed, the carrier injection scheme is employed to modulate the MRRs. As a proof of principle, a three-input reconfigurable logic circuit based on three cascaded carrier-injection MRRs is designed and fabricated, and several typical logic operations of three-operand with an operation speed of 100 *Mbps* are finally demonstrated successfully.

## Methods

### Device fabrication

**Figure 8:**
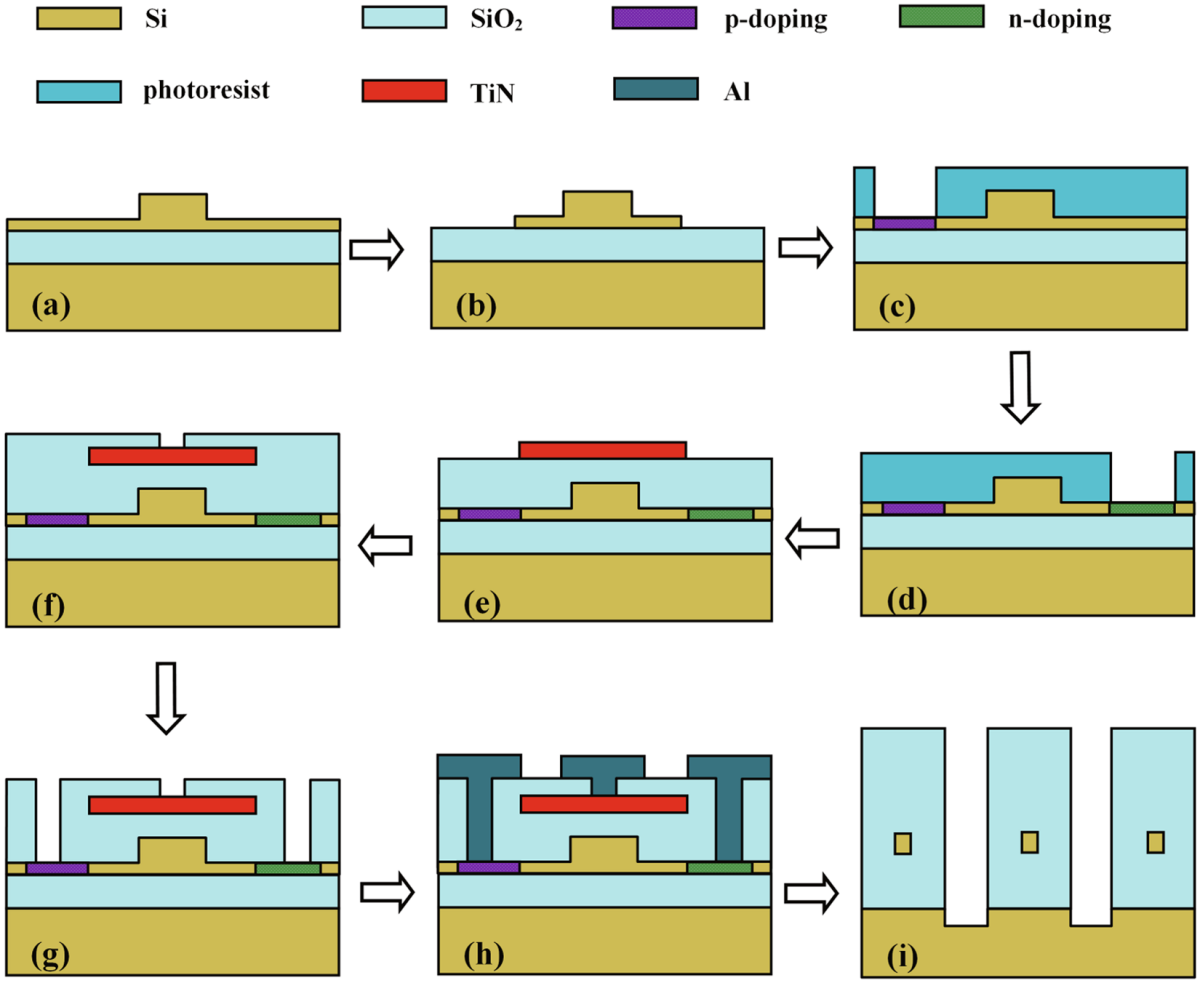
Processing flows of the device: (**a**,**b**) etching of the top Si layer by 150 nm and 70 nm, respectively, (**c**,**d**) p- and n- doping and through boron and phosphorus implantation, (**e**) deposition and etching of the TiN layer to form the microheater, (**f**,**g**) etching of the *SiO*_2_ layer to form the via holes to the PIN diodes and microheaters, (**h**) deposition and etching of the Al layer to form the wires and pads, (**i**) deep etching to form the end-face of the SSCs.

248-nm deep ultraviolet (UV) lithography is employed to define the waveguide patterns, and Inductively Coupled Plasma (ICP) is employed to etch the top silicon layer (Fig. 8(a,b)). The bus and ring waveguides are formed by a submicron rib waveguide with a height of 220 nm, a width of 400 nm, and a slab thickness of 70 nm, which only supports quasi-TE fundamental mode (the quasi-TE fundamental mode of the waveguide is shown in inset in Fig. 1(a)). The radii of MRRs are designed to be 10.00 *μm*, 10.03 *μm* and 10.06 *μm* in order to induce slightly different initial resonant wavelengths to the three MRRs. The gaps between the ring and straight waveguides are chosen to be 260 nm to achieve high extinction ratios. In order to enhance the coupling efficiency between the waveguide and the lensed fiber, the 200-*μm*-long linearly inversed nanotapers with 180-nm-wide tip are fabricated on the input and output terminals. Following the silicon waveguides are formed, the p- and n-doping regions with phosphorus and boron concentrations of 5.5 × 10^20^ *cm* ^−3^ are formed around ring waveguides to form PIN modulation structure which are employed to modulate MRRs. In order to achieve high modulation efficiency and low optical absorption loss, the edge-to-edge distance from the doped regions to the ring waveguides is designed about 500 nm (Fig. 8(c,d)). A 1.5-*μm*-thick *SiO*_2_ layer is deposited as the upper cladding layer using plasma-enhanced chemical vapor deposition (PECVD). After that, the 150-nm-thick TiN microheaters are fabricated on the top of MRRs using deep UV photolithography and dry etching (Fig. 8(e)) in order to control the resonant wavelengths of MRRs accurately. Via holes to PIN diodes and microheaters are formed through two step etching processes (Fig. 8(f,g)), and then a 1-*μm*-thick Al layer is sputtered and patterned to form printed tracks and pads to connect the PIN diodes and microheaters (Fig. 8(h)). Finally, the end-face of the SSC (spot size converter) is exposed by a 110-*μm*-deep etching process as the world-to-chip interface in order to improve the coupling efficiency between the SSC and lensed fiber (Fig. 8(i)).
